# Transcriptional Analysis of MexAB-OprM Efflux Pumps System of *Pseudomonas aeruginosa* and Its Role in Carbapenem Resistance in a Tertiary Referral Hospital in India

**DOI:** 10.1371/journal.pone.0133842

**Published:** 2015-07-29

**Authors:** Debarati Choudhury, Anupam Das Talukdar, Manabendra Dutta Choudhury, Anand Prakash Maurya, Deepjyoti Paul, Debadatta Dhar Chanda, Atanu Chakravorty, Amitabha Bhattacharjee

**Affiliations:** 1 Department of Life Science & Bioinformatics, Assam University, Silchar, Assam, India; 2 Department of Microbiology, Assam University, Silchar, Assam, India; 3 Department of Microbiology, Silchar Medical College and Hospital, Silchar, Assam, India; Cornell University, UNITED STATES

## Abstract

Carbapenem resistance presents severe threat to the treatment of multidrug resistant *Pseudomonas aeruginosa* infections. The study was undertaken to investigate the role of efflux pumps in conferring meropenem resistance and effect of single dose exposure of meropenem on transcription level of *mexA* gene in clinical isolates of *P*. *aeruginosa* from a tertiary referral hospital of India. Further, in this investigation an effort was made to assess whether different components of MexAB-OprM operon expresses in the same manner and the extent of contributions of those components in meropenem resistance in its natural host (*P*. *aeruginosa*) and in a heterologous host (*E*. *coli*). Out of 83 meropenem nonsusceptible isolates, 22 isolates were found to possess efflux pump activity phenotypically. Modified hodge test and multiplex PCR confirmed the absence of carbapenemase genes in those isolates. All of them were of multidrug resistant phenotype and were resistant to all the carbepenem drug tested. MexAB-OprM efflux pump was found to be overexpressed in all the study isolates. It could be observed that single dose exposure meropenem could give rise to trivial increase in transcription of *mexA* gene. Different constructs of MexAB-OprM (mexR-mexA-mexB-OprM; mexA-mexB-OprM; mexA-mexB) could be expressed in both its natural (*P*. *aeruginosa* PAO1) and heterologous host (*E*. *coli* JM107) but transcription level of *mexA* gene varied in both the hosts before and after single dose exposure of meropenem. Different components of the operon failed to enhance meropenem resistance in *E*. *coli* JM107 and *P*. *aeruginosa* PAO1. This study could prove that MexAB-OprM efflux pump can significantly contribute to meropenem resistance in hospital isolates of *P*. *aeruginosa* where an acquired resistant mechanism is absent. Thus, equal importance should be given for diagnosis of intrinsic resistance mechanism so as to minimize treatment failure. As meropenem could not enhance *mexA* transcriptions significantly, there might be a possibility that the increase in expression of efflux pump genes does not mediated by single antibiotic but rather by a combination of antipseudomonal drugs which are used during treatments. Early detection of efflux genes will help in selection of proper therapeutic options.

## Introduction

*Pseudomonas aeruginosa* pose severe threat in treatment of nosocomial infections because of its intrinsic and acquired resistance towards a wide range of antimicrobial agents. Carbapenems play a significant role in treatment of the infection caused by multidrug resistant *P*. *aeruginosa* but development of resistance towards this particular group of drug has further restricted the therapeutic options [1]. In absence of acquired resistant determinants (carbapenem hydrolyzing enzymes), carbapenem resistance in *P*. *aeruginosa* is mediated by various intrinsic mechanisms which includes increased activity of tripartite multi drug efflux pumps belonging to resistance-nodulation-cell division (RND) family (examples include MexAB-OprM; MexCD-OprJ; MexEF-OprN; MexXY-OprM; MexJK-OprM; MexVW-OprM); down regulation of outer membrane porin, OprD and increased production of chromosomally encoded beta-lactamases [2].

Overexpression of MexAB-OprM is generally mediated by mutation in a repressor gene, *mexR* (*nalB* mutant) located upstream of the MexAB-OprM operon [3]. The mutation leads to loss of DNA binding capacity of the mexR repressor protein to the mexR-mexA intergenic region and thereby losing its ability to repress MexAB-OprM operon. This results in hyperexpression of MexAB-OprM in *nalB* mutants [4,5]. *nalD* and *nalC* type of mutants have also been identified which occurs in response to mutation in *nalC* and *nalD* gene respectively. Both type of the mutants lead to upregulation of MexAB-OprM [6].

Efflux pump overexpression which results in subsequent reduction of antibiotic concentration allows the bacteria to select high level resistant determinants (production of carbapenemases). Because of its association with other resistance mechanisms, the diagnosis by phenotypic analysis often gets complicated because the high level acquired resistant determinants sometimes masks the contribution of overexpressed efflux on MICs. Efflux mediated resistance in clinical isolates can be detected more accurately by molecular methods [7]

Considering the impact of efflux pump mediated resistance mechanism on antimicrobial therapy in clinical settings and lack of data in this regard from this particular region of the world; the study was undertaken to investigate the role of efflux pump based mechanism conferring meropenem resistance among nosocomial isolates of *P*. *aeruginosa* collected from a tertiary referral hospital of north east India. Another problem with efflux mediated resistance is that they can be overexpressed during treatment which leads to treatment failure because in such situation, the antibiotics which were chosen on the basis of original susceptibility profiles no longer work. Therefore, with a view to study the effect of a carbapenem drug (meropenem) on expression of MexAB-OprM efflux pump, transcriptional levels of *mexA* gene was determined after single dose exposure of meropenem in efflux pump overexpressing strains. Further, in this study, different constructs of MexAB-OprM operon was cloned in its natural host (*P*. *aeruginosa*) and in a heterologous host (*E*. *coli*) to examine the expression pattern of different components of the operon in both the hosts and to evaluate their ability to mediate meropenem resistance.

## Methods

### Bacterial Isolates

A total of 83 carbapenem non susceptible clinical isolates of *P*. *aeruginosa* were obtained from the patients who were admitted to or attended the clinics of Silchar medical college and hospital, Assam, India from March, 2013 to February, 2014. Carbapenem nonsusceptible *P*. *aeruginosa* isolates were selected on the basis of resistance to meropenem. *P*. *aeruginosa* PAO1 (Kindly donated by Prof. Keith Poole, Queens University, Canada) was taken as the quality control strain.

### Ethical Approval

The work was approved by Institutional Ethical committee of Assam University, Silchar vide reference no.: IEC/AUS/2013-003. The authors confirm that participants provided their written informed consent to participate in this study. Consent process was approved by the institutional ethics committee.

### Phenotypic detection of efflux pump activity

Efflux pump activity of the strains were phenotypically detected by using meropenem (10 mg, Himedia, Mumbai) with and without CCCP (carbonyl cyanide m-chlorophenylhydrazone, 100mM, Himedia, Mumbai)[8].

MIC (Minimum inhibitory concentration) reduction assay was performed with meropenem alone and in combination with CCCP at concentration 20 μg/ml [9]. An efflux pump-overexpressed phenotype was defined as any strain exhibiting at least a fourfold decrease in MIC when tested in the presence of CCCP. *P*. *aeruginosa* PAO1 and *P*. *aeruginosa* knockout mutant (Kindly donated by Prof. Keith Poole, Queens University, Canada) of MexAB-OprM was taken as negative control.

### Detection of carbapenemases

Presence of carbapenemase activity in the selected isolates were assessed by modified Hodge Test [10]. To confirm the absence of carbapenemase genes, PCR assay was performed for detection of various carbapenemase gens, *bla*_VIM_, *bla*_NDM_, *bla*_IMP_, *bla*_OXA-48_, *bla*_OXA_-_23, -24/40_ and _-58_. The reaction conditions and primers were used as described previously [11, 12, 13, 14]

### Antimicrobial susceptibility testing and Minimum Inhibitory Concentration determination

Antibiotic susceptibility testing was performed on Mueller-Hinton agar (Himedia, Mumbai, India) plates by Kirby-Bauer disc diffusion method and interpreted as per CLSI recommendations [15]. The antibiotics tested viz., ciprofloxacin (5μg), amikacin (30μg), gentamicin (10μg), carbenicillin (10μg), polymixin B (300 μg), ceftazidime (30 μg), Piperacillin-Tazobactam (100/10 μg) (Himedia, Mumbai, India).

Minimum inhibitory concentration (MIC) was determined on Muller Hinton Agar plates by agar dilution method against imipenem and meropenem (Himedia, Mumbai, India) according to CLSI guidelines and interpreted according to CLSI breakpoint [15].

### Detection of transcription of MexAB-OprM by quantitative real time PCR before and after single dose exposure of meropenem

Expression of *mexA* gene was determined by qRT-PCR as described previously with some modification [16]. RNA was isolated from overnight culture grown to log phase of growth using RNeasy Kit (Qiagen, India). cDNA was prepared from mRNA using QuantiTect Reverse Transcription Kit (Qiagen, India). All the Preapared cDNA were subjected to semiquantitative PCR. mRNA transcription level was determined using SYBRGreen PCR Master Mix (Applied Biosystems, Foster City, CA) in Step one plus real time PCR system (Applied Biosystem, USA). Relative quantities of gene expression were calculated using the ΔΔCt method. Expression of the 30S ribosomal gene *rpsL* was determined in parallel to normalize the transcriptional levels of target genes and they were further calibrated against corresponding mRNA expression by *P*. *aeruginosa* PAO1. Samples were run in triplicate and contained 50 ng of RNA per reaction. MexAB-OprM efflux pump was considered overexpressed when transcription level was atleast 2-fold higher compared with that of *P*. *aeruginosa* PAO1 [1].

Transcription levels were also determined after antibiotic exposure by growing in Luria Bertani (LB) Broth containing 0.125 meropenem ug/ml. All experiments were carried out in triplicates.

### Cloning and analysis of MexAB-OprM efflux pump system in *E*. *coli* and in *P*. *aeruginosa*

Different components of MexAB-OprM operon was cloned in a heterologous host *Escherichia coli* and in natural host *P*. *aeruginosa*.

**Fig 1:**
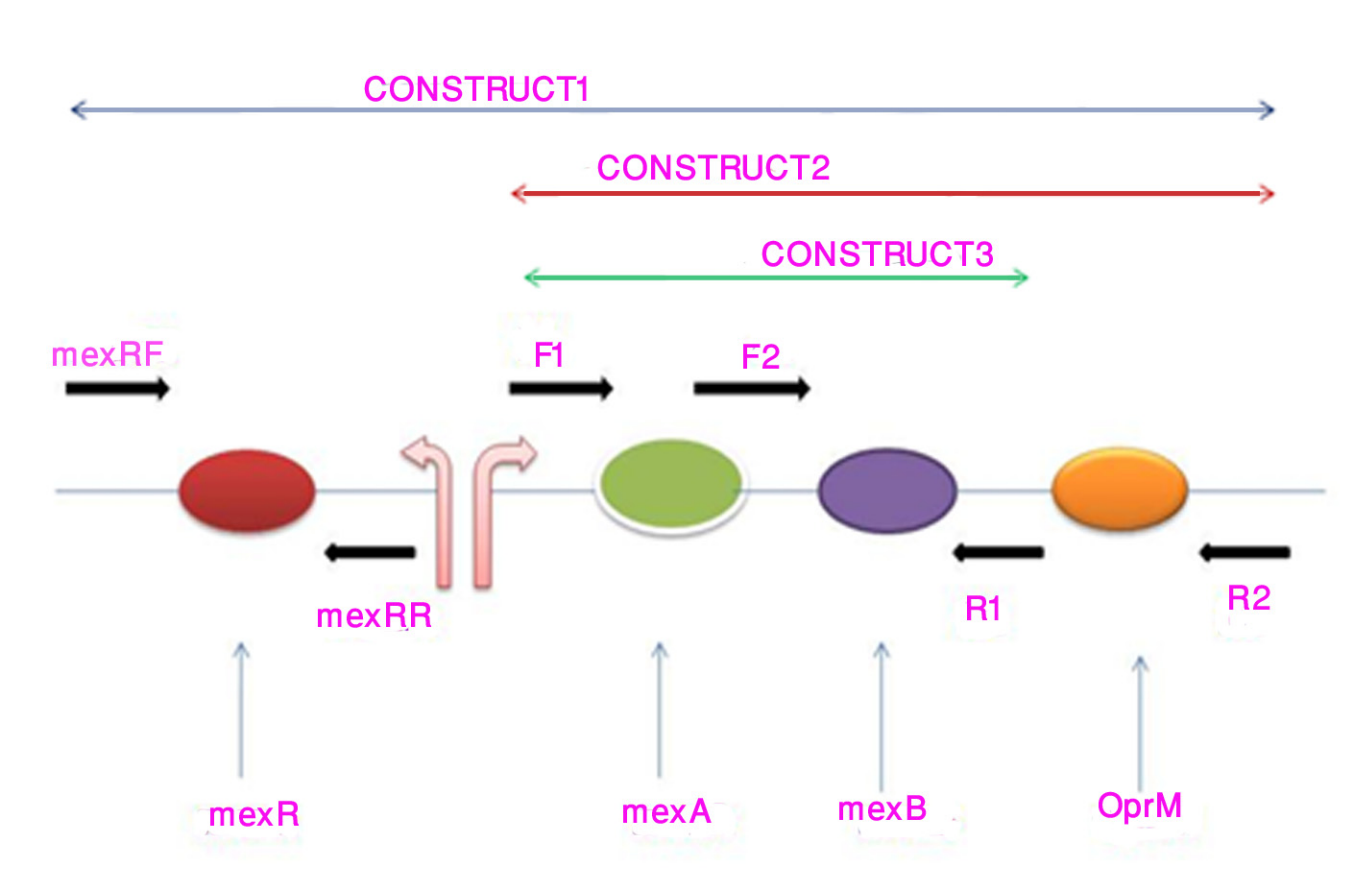
Figure describing different regions of mexAB-OprM operon which are amplified using different primers as shown.

For cloning, three different constructs were made which are given as follows: Construct 1: MexAB; Construct 2: MexAB-OprM; Construct 3: MexR+MexAB-OprM (Fig 1). Primers used for amplification are designed for the study and are in given in Table 1. PCR amplifications were performed using 50 μl of total reaction volume. Reactions were run under the following conditions: initial denaturation at 94°C for 2 min, 32 cycles of 94°C for 25 Sec., 52°C for 1min, 72°C for 3min and final extension at 72°C for 10 min. PCR products were examined on 1.0% (w/v) agarose gels and purified using the GeneJET Gel extraction Kit (Thermo Scientific). Amplified products were ligated in P^GEM-T^ vector (Promega) and were transformed on *E*.*Coli* JM107 and *P*. *aeruginosa* PAO1 by heat shock method. Transformants were further selected on screen agar.

**Table 1:** Details of primers used for cloning of efflux pump genes.

Construct	Construct1 (MexR+MexAB)	Construct2 (MexAB-OprM)	Construct3 (MexAB)
Primer name and sequence	mexR F: 5’ AGTCTTGGACTTTGTTCCAAC 3’	F1: 5’ GCATCAGGTCGGGATTCACGG 3’	F1:5’GCATCAGGTCGGGATTCACGG 3’
	R2: 5’ TAGGGTCGGCGTTCTTGCATGG 3’	R2: 5’ TAGGGTCGGCGTTCTTGCATGG 3’	R1: 5’ GGACAGAACGACAGCGGTA 3’
Amplicon length (bp)	6505	6231	4661

Antibiotic stress and determination of transcription level of mexA gene in *E*.*coli* JM107 and *P*. *aeruginosa* PA01, both carrying MexAB-OprM or its components were done in the same procedure as described above.

MIC of the transformants (*E*.*coli* JM107 and *P*. *aeruginosa* PA01) carrying MexAB-OprM or its components were tested against meropenem.

### DNA fingerprinting by REP PCR

REP PCR was performed to determine clonal relatedness of all the isolates selected under the present work. The primers and reaction condition used were same as described previously [17].

## Results

### Phenotypic screening of efflux pump activity and detection of carbapenemases

Among 83 carbapenem nonsucceptible isolates 45% (38/83) were found to exhibit enhanced efflux pump activity against meropenem. 26% of them (22/83) were devoid of carbapenemase activity and multiplex PCR could not establish the presence of carbapenemase genes in the isolates. All the 22 carbapenem nonsusceptible and carbapenemase nonproducing isolates were found to demonstrate efflux pump activity phenotypically and a sharp reduction in MIC was observed in all the isolates when CCCP was added at a fixed concentration of 20 ug/ml (Table 2).

**Table 2:** MIC reduction assay using meropenem.

Sl No.	Isolate ID	MIC against Meropenem (μg/ml)	MIC against Meropenem (after addition of CCCP) (μg/ml)	Fold decrease in MIC
1	AM-361	8	0.25	32
2	AM-18	8	0.5	16
3	AM-121	16	2	8
4	AM-329	16	2	8
5	AM-219	16	0.5	32
6	AM-173	16	4	4
7	AM-529	32	8	4
8	AM- 592	32	8	4
9	AM-335	32	2	16
10	AM-609	32	8	4
11	AM-131	32	4	8
12	AM-146	32	4	8
13	AM-67	64	16	4
14	AM-534	64	8	8
15	AM-536	64	4	16
16	AM-335	64	8	8
17	AM-75	64	8	8
18	AM-608	128	32	4
19	AM-466	128	16	8
20	AM-326	128	32	4
21	AM-352	128	32	4
22	AM-64	128	32	4

### Antimicrobial susceptibility testing and Minimum Inhibitory Concentration determination

All the isolates with phenotypically predicted efflux pump activity were of multidrug resistant phenotype. However, Polymixin B was found to have moderate activity. Other group of antibiotics came up with very low efficacy (Table 3) The isolates were above breakpoint level for the all the carbapenem drug tested (Table 4).

**Table 3:** Antibiogram of *P*. *aeruginosa* with increased efflux pump activity.

Antibiotics	Total no. of isolates with increased efflux pump activity	No. of susceptible samples
Amikacin	22	1
Gentamycin	22	3
Pipericillin-tazobactum	22	5
Faropenem	22	3
Polymixin b	22	9
Carbenicillin	22	3
Ceftazidime	22	4
Tigicycline	22	6
Ciprofloxacin	22	4

**Table 4:** MIC_50_ and MIC_90_ of efflux mediated carbapenem resistant *P*. *aeruginosa* isolates.

Antibiotics	MIC_50_	MIC_90_
Imipenem	16 μg/ml	128 μg/ml
Meropenem	32 μg/ml	128 μg/ml
Ertapenem	64 μg/ml	>256 μg/ml

### Detection of transcription of MexAB-OprM by quantitative real time PCR before and after single dose exposure of meropenem

**Fig 2:**
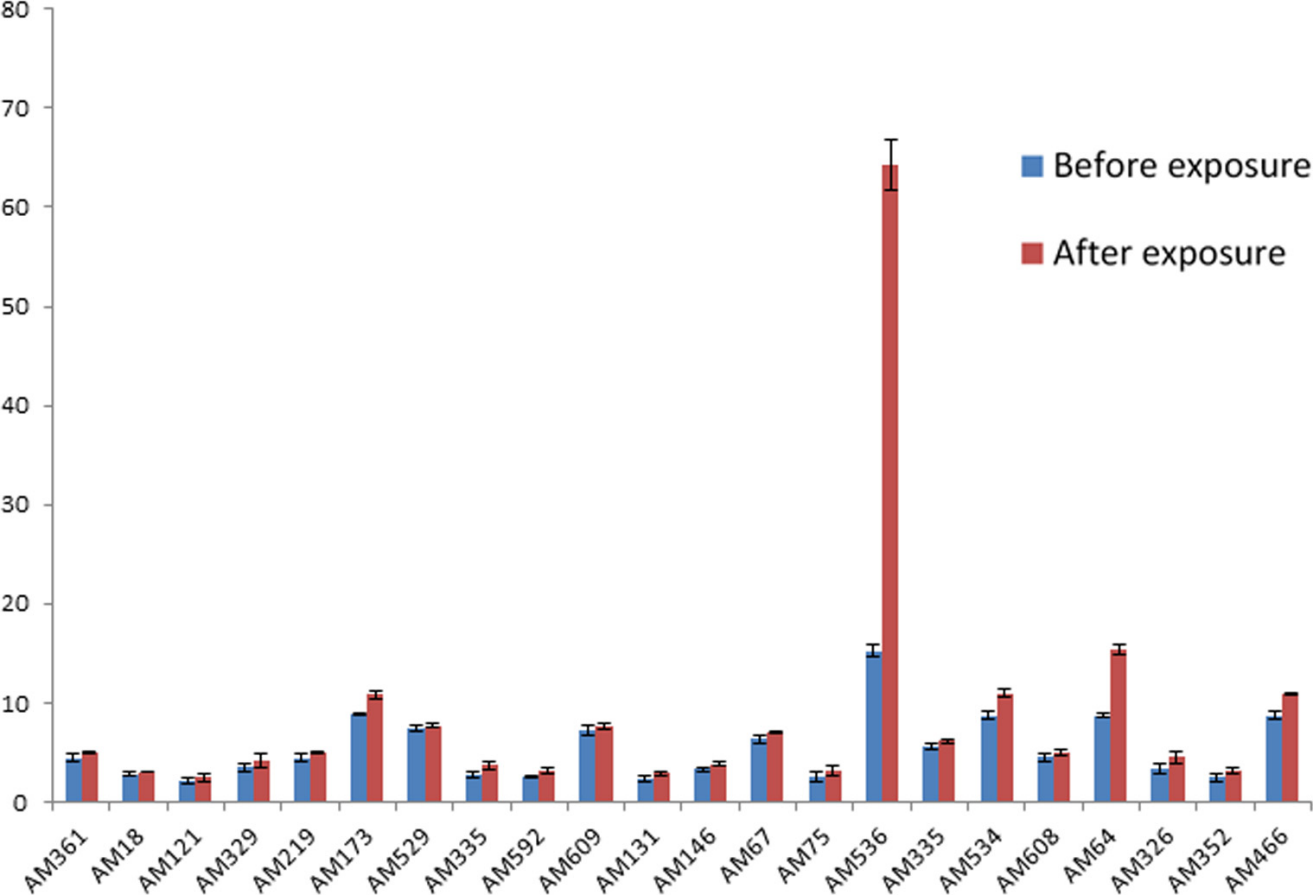
Expression of *mexA* gene before and after single dose exposure with meropenem in bacteria.

As the test isolates with high MIC value were also devoid of carbapenemase activity, so the expression of MexAB-OprM efflux pump was investigated in the isolates from each MIC range. Studies revealed that all the 22 isolates belonging overexpressed MexAB-OprM efflux pump. Further, to check the effect of a commonly prescribed carbapenem, meropenem on the expression of MexAB-OprM, the transcriptome level of *mexA* gene was determined in the same strains after giving single dose exposure with meropenem. Expression of mRNAs from bacteria with and without antibiotic exposure are shown in Fig 2. It can be seen that, though there is slight increase in expression level of *mexA* gene for most of the isolates but the increase is not significant. However, in case of two isolates there is a significant change in expression level of *mexA*; approximately 4 fold and 2 fold increase in expression can be seen for AM536 and AM64 respectively.

### Cloning and analysis of MexAB-OprM efflux pump system in *E*. *coli* and in *P*. *aeruginosa*

**Fig 3:**
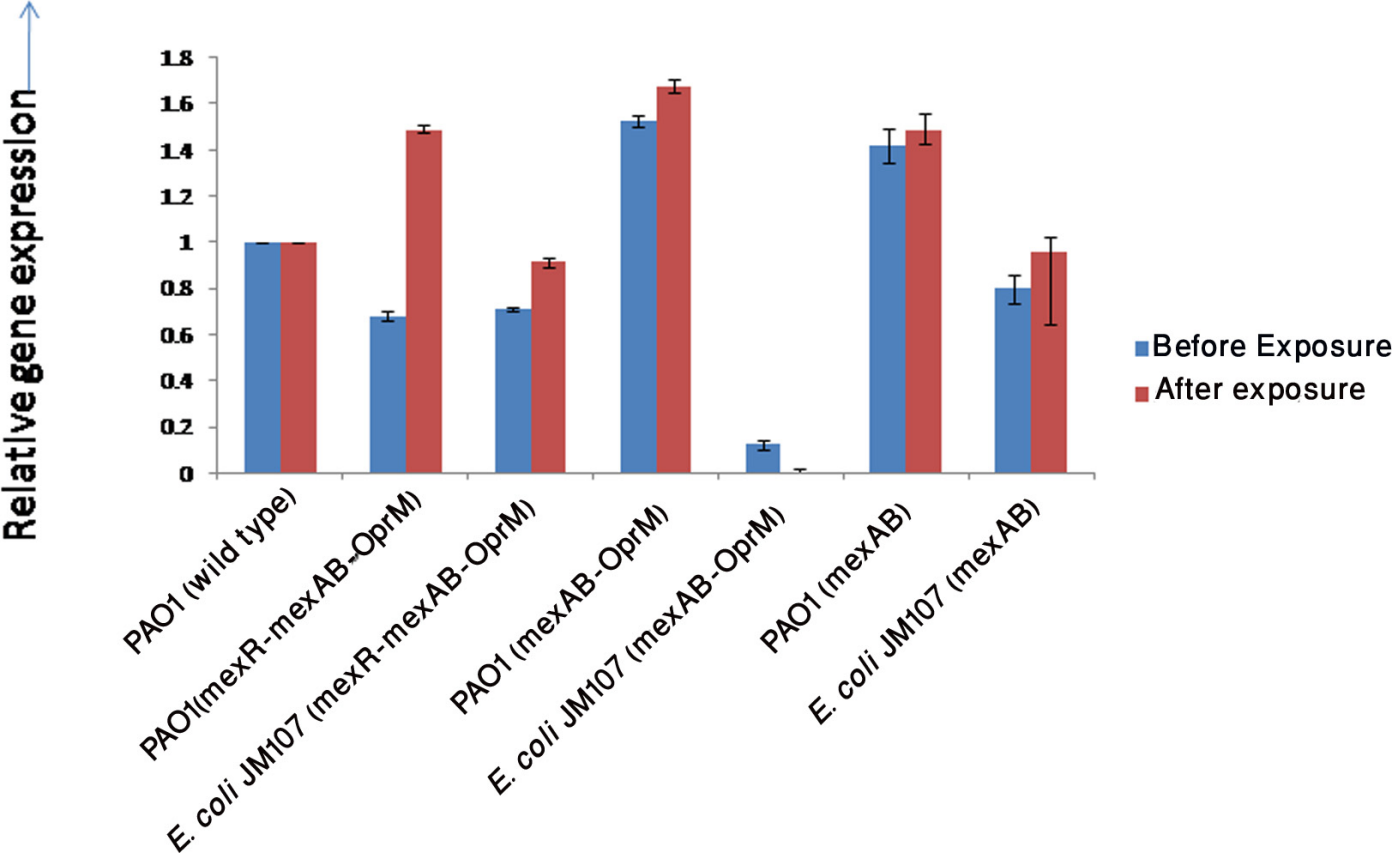
Expression of *mexA* gene before and after single dose exposure in transformants.

In order to study the expression of different components of MexAB-OprM operon in the natural host and in a non-host, different components of the operon (Construct 1, 2 and 3) were expressed in *P*. *aeruginosa* PAO1 and in *E*. *coli* JM107 respectively. The expression of *mexA* gene was checked in transformants. Expression of *mexA* gene in transformants (*E*. *coli* JM107 and *P*. *aeruginosa* PAO1) before and after drug exposure carrying different components of MexAB-OprM is shown in Fig 3. In PAO1 carrying construct 1 (mexR-mexA-mexB-OprM), the expression level of *mexA* gene was comparatively lower than that of *E*.*coli* JM107 carrying the same construct. However, after exposure, the increase in *mexA* level in *E*. *coli* was much lower compared to PAO1. In PAO1 carrying construct 2 (MexAB-OprM), *mexA* expression was higher than *E*.*coli* JM107 carrying the same construct. Though after antibiotic stress, a completely different scenario was observed. Exposure resulted in slight increase in *mexA* level in PAO1 carrying construct 2, but in case *E*.*coli* JM107 with construct 2, it resulted in decrease in expression of *mexA*. *E*.*coli* JM107 with construct 3 (MexAB) expressed *mexA* in lower level than that of PAO1 with same construct and were around in the same level as that of *E*. *coli* and *P*. *aeruginosa* carrying construct 3 respectively and antibiotic stress did not result in rise in transcript level. In order to examine, how the components contribute to carbapenem resistance, their MIC against meropenem was checked. It was seen that regardless of the expression of MexAB-OprM in the transformants, they failed to enhance meropenem resistance in *E*. *coli* JM107 and *P*. *aeruginosa* PAO1.

### DNA fingerprinting by REP PCR

**Fig 4:**
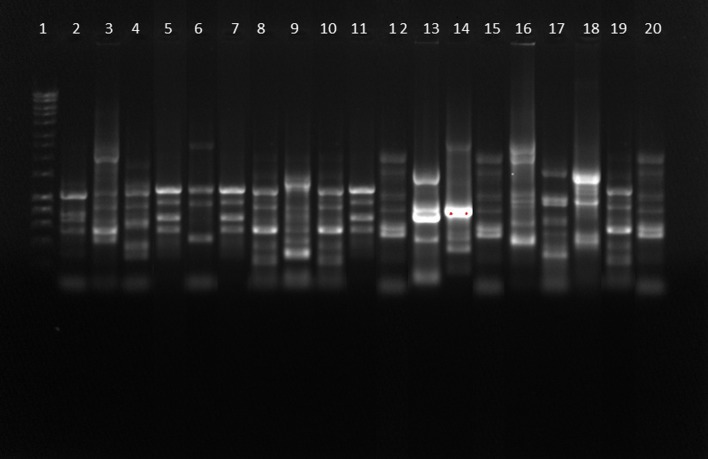
REP PCR showing different REP types of *P*. *aeruginosa* possessing increased efflux pump activity. Lane 1: Hyper ladder (1 kb); lane 2 to 20: test samples. 13 REP types can be seen in Lane 2, 3, 4, 5, 6, 8, 9, 12, 13, 14, 16, 17 and 18.

A total of 13 REP type was found and no significant similarity between the study isolates could be observed among the study isolates (Fig 4).

## Discussion

The grave situation of pan resistance in *P*. *aeruginosa* has been observed globally in the last decade. The decreasing effectiveness of some antimicrobials limits the therapeutic options to a great extent [1]. Carbapenems are the most effective agents used in the treatment of multi drug resistant *P*. *aeruginosa* infections but their effectiveness is significantly reduced due to development of bacterial resistance. [8] Carbapenem resistance in *P*. *aeruginosa* often associated with resistance to other antimicrobial classes which complicates the selection of appropriate antimicrobial chemotherapy [18]. The main mechanisms which leads to carbapenem resistance in *P*. *aeruginosa* includes production of carbapenem hydrolyzing enzymes (carbapenemases), reduced expression of outer membrane porin, OprD and overexpression of efflux pumps belonging to RND family. Intrinsically produced efflux pumps play a major role in carbapenem resistance [19]. The RND efflux pump systems subsists as a tri-partite system consisting of a cytoplasmic exporter protein (RND) and an outer membrane factor (OMF) and a membrane fusion protein spanning the periplasm (MFP) that link RND and OMF[3]. Among the ten RND efflux pumps identified in *P*. *aeruginosa*, MexAB-OprM is one of the most clinically important pumps and contributes significantly to intrinsic resistance towards a wide range of antimicrobials including quinolones, macrolides, chloramphenicol, tetracycline, novobiocin, most beta-lactams including meropenem but not imipenem [4].

In this study, carbapenem (Imipenem and meropenem) MIC was above breakpoint level for all the 22 carbapenem nonsusceptible carbapenemase nonproducing isolates. They were resistant to all the tested antimicrobials and overproduces MexAB-OprM system. A strong correlation was observed between meropenem resistance and mexA overexpression which is in coincidence with other previous reports [10].

The isolates with overexpressed MexAB-OprM were given single dose exposure by meropenem to investigate its effect on transcriptional level of *mexA* gene. Despite being a preferred substrate of MexAB-OprM pump,[3] meropenem leads to a slight increase in mRNA level of *mexA* gene except two strains (AM536 and AM64), where a significant change in expression level was observed after induction. The relative increase in mRNA level of *mexA* gene varies among isolates. However, transcription level of MexAB-OprM could not be correlated with respective MIC value of the isolates towards meropenem antibiotics. Roiu *et al*. [20] while examining the effect of antibiotic treatment on expression of RND efflux pump gene found that during treatment the frequency of efflux genes (*mexA* and *mexX*) increases. A significant increase in frequency was observed when all the genes were considered globally and the most used antimicrobials were piperacillin–tazobactam (26 pts), amikacin (22 pts), meropenem (20 pts), cefepime (19 pts), and ciprofioxacin (6pts), but in combination for 69% of pts. Correlation between overexpression of a specific gene and corresponding administered antibiotic could not be observed [20]. In this study also, it was seen that, meropenem failed to increase *mexA* transcription significantly. This predicts that increase in expression of efflux pump genes does not mediated by single antibiotic but rather by a combination of antipseudomonal drugs which are used during treatments. This particular scenario points towards urgent need for early detection of efflux genes which will help in selection of appropriate therapeutic agents.

Expression of MexAB-OprM efflux pump is regulated mainly by a gene *mexR* located upstream of the mexAB-OprM operon which encodes a repressor that binds as a homodimer within the mexR-mexA intergenic region overlapping *mexR* and *mexA* promoters which ultimately represses transcription of mexAB-OprM operon and negatively autoregulates its own transcription [3,5]. Mutations in *mexR* gene (*nalB* mutants) leads to mexAB-oprM hyperexpression [6]. Hyperproduction may also results from mutation in recently identified genes PA3721 (*nalC*) and PA3574 (*nalD*) [3]. With a view to study how different components of mexAB-OprM operon expresses in a heterolous host *E*. *coli* and how the components contributed to carbapenem resistance in a natural host and in a non-host, different components of mexAB-OprM (Fig 1) were cloned in its host *P*. *aeruginosa* PAO1 and in non-host *E*. *coli* JM107 and their role towards meropenem resistance was assessed.

In PAO1 carrying construct 1 (mexR-mexA-mexB-OprM), the expression level of *mexA* gene was comparatively lower than that of *E*.*coli* JM107 carrying the same construct. One possible explanation for this would be the presence of two repressor gene, *mexR*. Similar results were obtained previously when it was tried to assess the expression of *mexA* gene in a *mexR* deficient mutant (K758). Inactivation of *mexR* resulted in a two fold increase in *mexA-lacZ* fusion expression compared to its *mexR+* parent strain. In addition to increased expression of the efflux operon, the mutant exhibited increased resistance towards tetracycline, chloramphenicol, naladixic acid, ciprofloxacin and cefotaxime [21]. In this study, on the other hand, *E*.*coli* JM107 carrying construct 1 (mexR-mexA-mexB-OprM) did not show any resistnace towards meropenems. In *P*. *aeruginosa* carrying construct 2 (mexA-mexB-OprM), expression of *mexA* was higher than that of *E*.*coli* JM107 carrying the same construct. This is however obvious as MexAB-OprM system is intrinsically present in *P*. *aeruginosa* [22]. *P*. *aeruginosa* with an extra set of plasmid borne efflux operon (PAO1 with construct 2) failed to enhance meropenem MIC. Even though the operon is functional in *E*. *coli*, but it could not demonstrate meropenem resistance. A previous study reported that, *E*. *coli* DH5α carrying MexAB-OprM operon failed to enhance antibiotic resistance towards a series of antimicrobials including detergents, dyes, quinolones, macrolides, chloramphenicol and penicillin subgroup of beta-lactams [23]. However, a acrAB deleted strain of *E*. *coli* exhibited elevated resistance to the antimicrobials mentioned above. Though AcrAB efflux system of *E*. *coli* shares a considerable homology with the MexAB of *P*. *aeruginosa*, but the resistance level is much narrower in case of *E*. *coli* expressing MexAB-OprM than in *P*. *aeruginosa*. [23]. The expression of *mexA* gene in *E*.*coli* with construct 3 (MexAB) was lower than that of PAO1 with same construct and were approximately in the same level as that of *E*. *coli* and *P*. *aeruginosa* carrying construct 2 respectively without any sort of augmentation in resistance. The present study reports that meropenem resiatance cannot be attributed by *E*. *coli* carrying components of MexAB-OprM operon.

Antibiotic exposure of transformants (*E*. *coli* JM107 and *P*. *aeruginosa* PAO1) with meropenem revealed interesting results. After antibiotic stress, transcript level of *mexA* in PAO1 with construct 1 was increased in comparison to *E*. *coli* with same construct. In case of construct 2, there was trivial increase in transcription in PAO1 but in *E*. *coli*, there was an unexpected decrease in transcript level. A detailed study is required to understand the mechanism behind this scenario. Construct 3 resulted in no significant change in expression of mexA after exposure. A previous study demonstrated that imipenem and meropenem induction leads to varied responses in *OprM* and *OprN* efflux genes. Though, in most cases imipenem and meropenem increased *OprM* and *OprN* mRNA levels. The authors concluded that responses of antibiotic resistant genes in *P*. *aeruginosa* is to some extent frenzied [24]. The present study also, above results calls for further investigation in mRNA regulatory pathways of MexAB-OprM efflux pump genes as far as meropenem resistance is concerned.

## Conclusion

In the present study it has been observed that in absence of acquired resistant determinants (carbapenemases), overexpression of MexAB-OprM efflux pumps was responsible for meropenem resistance in clinical isolates of *P*. *aeruginosa* in this geographical region of the world. This study also establishes the fact that combination of factors might be responsible to induce overexpression of MexAB-OprM and despite expression of different components of MexAB-OprM in a heterlogous host *E*. *coli*, the components failed to enhance meropenem resistance in both host and in non host. In spite of being a well characterized mechanism of resistance, MexAB-OprM mediated mechanism is often neglected and diagnostic laboratories are yet to incorporate a phenotypic tool for their detection in routine practice. Thus, equal importance should be given for diagnosis of intrinsic resistance mechanism so as to minimize treatment failure. As meropenem could not enhance mexA transcriptions significantly, there might be a possibility that the increase in expression of efflux pump genes does not mediated by single antibiotic but rather by a combination of antipseudomonal drugs which are used during treatments. The study emphasizes early detection of this intrinsic resistance mechanism improving the selection of proper therapeutic options.

## Supporting Information

S1 Table Clinical details of *P*. *aeruginosa* isolates with overexpressed *mexA* gene. (DOCX)

## References

[1] Xavier ED, Picao CR, Girardello R, Fehlberg CCL, Gales CA. Efflux pumps expression and its association with porin down-regulation and b-lactamase production among *Pseudomonas aeruginosa* causing bloodstream infections in Brazil. BMC Microbiol. 2010; 10:217–223. 10.1186/1471-2180-10-217 20704733 PMC2927533

[2] Pai H, Kim JW, Kim J, Lee JH, Choe KW, Gotoh N. Carbapenem Resistance Mechanisms in *Pseudomonas aeruginosa* Clinical Isolates. Antimicro Agents Chemother. 2001; 45(2):480–484. 10.1128/AAC.45.2.480-484.2001 PMC90316 11158744

[3] Evans K, Adewoye L, Poole K. MexR repressor of the *mexABoprM* multidrug efflux operon of *Pseudomonas aeruginosa*: identification of MexR binding sites in the *mexA*-*mexR* intergenic region. J Bacteriol. 2001; 183: 807–812. 11208776 10.1128/JB.183.3.807-812.2001 PMC94945

[4] Saito K, Eda S, Maseda H, Nakae T. Molecular mechanism of MexR-mediated regulation of MexAB-OprM efflux pump expression in *Pseudomonas aeruginosa* . FEMS Microbiol Lett. 2001; 195:23–28. 11166990 10.1111/j.1574-6968.2001.tb10492.x

[5] Poole K, Tetro K, Zhao Q, Neshat S, Heinrichs DE, Bianco N. Expression of the multidrug resistance operon *mexA-mexB-oprM* in *Pseudomonas aeruginosa*: *mexR* encodes a regulator of operon expression. Antimicrob Agents Chemother. 1996; 40:2021–2028. 8878574 10.1128/aac.40.9.2021 PMC163466

[6] Lister PD, Wolter DJ, Hanson ND. Antibacterial-Resistant *Pseudomonas aeruginosa*: Clinical Impact and Complex Regulation of Chromosomally Encoded Resistance Mechanisms. Clin Microbiol Reviews. 2009; 22 (4): 582–610. 10.1128/CMR.00040-09 PMC2772362 19822890

[7] Avrain L, Mertens P, Van Bambeke F. RND efflux pumps in *P* *aeruginosa*: underestimated underestimated resistance mechanism. CLI 2013, 26–28. Available: http://www.uclouvain.be/cps/ucl/doc/ir-ldri/images/Avrain-2013-1.pdf.

[8] Quale J, Bratu S, Landman D, Heddurshetti R. Molecular Epidemiology and Mechanisms of Carbapenem Resistance in *Acinetobacter baumannii* Endemic in New York City. Clinical Inf Diseases. 2003; 37:214–220. 10.1086/375821 12856214

[9] Kriengkauykiat J, Porter E, Lomovskaya O, Wong-Beringer A. Use of an Efflux Pump Inhibitor To Determine the Prevalence of Efflux Pump-Mediated Fluoroquinolone Resistance and Multidrug Resistance in *Pseudomonas aeruginosa* . Antimicrob Agents Chemother. 2005; 49(2):565–570. 15673734 10.1128/AAC.49.2.565-570.2005 PMC547318

[10] Tomas M, Doumith M, Warner M, Turton JF, Beceiro A, Bou G et al Efflux Pumps, OprD Porin, AmpC β-Lactamase, and Multiresistance in *Pseudomonas aeruginosa* Isolates from Cystic Fibrosis Patients. Antimicrob Agents Chemother. 2010; 54 (5): 2219–2224. 10.1128/AAC.00816-09 20194693 PMC2863613

[11] Yong D, Toleman MA, Giske CG, Cho HS, Sundman K, Lee K, et al Characterization of a New Metallo-β-Lactamase Gene, *bla*NDM-1, and a Novel Erythromycin Esterase Gene Carried on a Unique Genetic Structure in *Klebsiella pneumoniae* Sequence Type 14 from India. Antimicrob Agents Chemother. 2009; 53:5046–5054. 10.1128/AAC.00774-09 19770275 PMC2786356

[12] Y JH, Yi K, Lee H, Yong D, Lee K, Kim JM et al Molecular characterization of metallo-β-lacatamase-producing *Acinetobacter baumannii* and *Acinetbacter genomospecies* 3 from Korea: identification of two new integrons carrying the *blaVIM-2* gene cassettes. J Antimicrob Chemother. 2002; 49:837–840 12003980 10.1093/jac/dkf043

[13] Shibl A, Al-Agamy M, Memish Z, Senok A, Khader SA, Assiri A. The emergence of OXA-48 and NDM-1-positive *Klebsiella pneumoniae* in Riyadh, Saudi Arabia. Int. J. Infect. Diseases. 2013; 17: 1130–33. 10.1016/j.ijid.2013.06.016 24021566

[14] Mendes RE, Bell JM, Turnidge JD, Castanheira M, Jones RN. Emergence and widespread dissemination of OXA-23, -24/40 and -58 carbapenemases among *Acinetobacter* spp. in Asia-Pacific nations: report from the SENTRY Surveillance Program. Antimicrob. Chemother. 2009; 63: 55–59. 10.1093/jac/dkn434 18957398

[15] Clinical and Laboratory Standards Institute. Performance Standards for Antimicrobial Susceptibility Testing; Twenty-First Informational Supplement. M100-S21. CLSI, Wayne, PA, USA, 2011.

[16] Mesaros N, Glupczynski Y, Avrain L, Caceres NE, Tulkens PM, Van Bambeke F. Combined phenotypic and genotypic method for the detection of Mex efflux pumps in *Pseudomonas aeruginosa* . J Antimicrob Chemother. 2007; 59:378–386. 17289770 10.1093/jac/dkl504

[17] Versalovic J, Koueth T, Lupski JR. Distribution of repetitive DNA sequences in eubacteria and application to fingerprinting of bacterial genomes. Nucleic Acid Res. 1991; 19: 6823–6831. 1762913 10.1093/nar/19.24.6823 PMC329316

[18] Meletis G, Exindari M, Vavatsi N, Sofianou D, Diza E. Mechanisms responsible for the emergence of carbapenem resistance in *Pseudomonas aeruginosa* . Hippokratia 2012; 16(4): 303–307. 23935307 PMC3738602

[19] Livermore DM. Of *Pseudomonas*, porins, pumps and carbepenms. Antimicrob Agents Chemother. 2001; 47, 247–250. 10.1093/jac/47.3.247 11222556

[20] Riou M, Avrain L, Garch EL, Glupczynski Y, Pirnay JP, De Vos D et al. Influence of antibiotic treatments on gene expression of RND efflux pumps in successive isolates of *Pseudomonas aeruginosa* collected from patients with nosocomial pneumonia hospitalized in Intensive Care Units from Belgian Teaching Hospitals. ECCMID, 10–13 April 2010, Vienna, Austria. P780.

[21] Poole K, Tetro K, Zhao Q, Neshat S, Heinrichs DE, Bianco N. Expression of the Multidrug Resistance Operon *mexA-mexB-OprM* in *Pseudomonas aeruginosa*: *mexR* Encodes a Regulator of Operon Expression. Antimicrobial Agents and Chemother. 1996; 40 (9): 2021–2028. 10.1128/aac.40.9.2021 PMC163466 8878574

[22] Poole K, Srikumar R. Multidrug efflux in *Pseudomonas aeruginosa*: components, mechanisms, and clinical significance. Curr Top Med Chem. 2001; 1:59–71. 11895293 10.2174/1568026013395605

[23] Srikumar R, Kon T, Gotoh N, Poole K. Expression of *Pseudomonas aeruginosa* Multidrug Efflux Pumps MexA-MexB-OprM and MexC-MexD-OprJ in a Multidrug-Sensitive *Escherichia coli* Strain. Antimicrobial Agents and Chemother. 1998; 42 (1): 65–71. 10.1128/aac.42.1.65 PMC105457 9449262

[24] Kolayli F, Karadenizli A, Savli H, Ergen K, Hatirnaz O, Balikci E et al Effect of carbapenems on the transcriptional expression of the oprD, oprM and oprN genes in*Pseudomonas aeruginosa* . J of Med Microbiol. 2004; 53: 915–920. 15314200 10.1099/jmm.0.45692-0

