# Supplementary material for: Transcriptional Analysis of MexAB-OprM Efflux Pumps System of Pseudomonas aeruginosa and Its Role in Carbapenem Resistance in a Tertiary Referral Hospital in India
Source: PLoS One. 2015 Jul 29;10(7):e0133842. doi: 10.1371/journal.pone.0133842 (PMC4519154; doi:10.1371/journal.pone.0133842)
Supplement: S1 Table — (DOCX) [file pone.0133842.s001.docx]

**S1 Table: Clinical details of *P. aeruginosa* isolates with overexpressed *mexA* gene.**

| SL No. | Sample ID | Sex | Age (Years) | Ward/OPD | Type of clinical specimen |
| --- | --- | --- | --- | --- | --- |
| 1 | AM-361 | Male | 40 | Surgery | urine |
| 2 | AM-18 | Female | 19 | Surgery | pus |
| 3 | AM-121 | Male | 14 | Orthopedics | Pus |
| 4 | AM-329 | Male | 50 | Surgery | Pus |
| 5 | AM-219 | Female | 45 | Female burn Unit | Pus |
| 6 | AM-173 | Male | 26 | Orthopedics | Pus |
| 7 | AM-529 | Male | 13 | Medicine | Urine |
| 8 | AM- 592 | Female | 45 | Ear Nose Throat | Oral swab |
| 9 | AM-335 | Female | 21 | Surgery | Pus |
| 10 | AM-609 | Male | 70 | Surgery | Pus |
| 11 | AM-131 | Female | 40 | Gynecology OPD | Urine |
| 12 | AM-146 | Male | 35 | Medicine | Urine |
| 13 | AM-67 | Male | 14 | Medicine | Urine |
| 14 | AM-534 | Female | 22 | Medicine | Urine |
| 15 | AM-536 | Male | 29 | Medicine | Urine |
| 16 | AM-335 | Female | 21 | Surgery | Pus |
| 17 | AM-75 | Female | 63 | Orthopedics | Pus |
| 18 | AM-608 | Male | 12 | Pediatrics | Urine |
| 19 | AM-466 | Female | 45 | Medicine | Urine |
| 20 | AM-326 | Male | 3 moths | Pediatrics | Urine |
| 21 | AM-352 | Female | 40 | Gynecology OPD | Urine |
| 22 | AM-64 | Female | 22 | Surgery | Pus |
